# Oral treatment of human gut microbiota associated IL-10^−/−^ mice suffering from acute campylobacteriosis with carvacrol, deferoxamine, deoxycholic acid, and 2-fucosyl-lactose

**DOI:** 10.3389/fmicb.2024.1290490

**Published:** 2024-01-25

**Authors:** Soraya Mousavi, Minnja S. Foote, Ke Du, Rasmus Bandick, Stefan Bereswill, Markus M. Heimesaat

**Affiliations:** Gastrointestinal Microbiology Research Group, Institute of Microbiology, Infectious Diseases and Immunology, Charité – Universitätsmedizin Berlin, Corporate Member of Freie Universität Berlin, Humboldt-Universität zu Berlin, and Berlin Institute of Health, Berlin, Germany

**Keywords:** natural compounds, *Campylobacter jejuni*, immune-modulatory effects, secondary abiotic IL-10^−/−^ mice, human gut microbiota associated mice, campylobacteriosis model, host-pathogen interaction, placebo-controlled preclinical intervention study

## Abstract

Food-borne *Campylobacter jejuni* infections constitute serious threats to human health worldwide. Since antibiotic treatment is usually not indicated in infected immune-competent patients, antibiotic-independent treatment approaches are needed to tackle campylobacteriosis. To address this, we orally applied carvacrol, deferoxamine, deoxycholate, and 2-fucosyl-lactose either alone or all in combination to human microbiota-associated IL-10^−/−^ mice from day 2 until day 6 following oral *C. jejuni* infection. Neither treatment regimen affected *C. jejuni* loads in the colon, whereas carvacrol lowered the pathogen numbers in the ileum on day 6 post-infection (p.i.). The carvacrol and combination treatment regimens resulted in alleviated diarrheal symptoms, less distinct histopathological and apoptotic epithelial cell responses in the colon, as well as diminished numbers of colonic neutrophils and T lymphocytes on day 6 p.i., whereas the latter cells were also decreased upon deferoxamine, deoxycholate, or 2-fucosyl-lactose application. Remarkably, the carvacrol, deferoxamine, and combination treatment regimens dampened *ex-vivo* IFN-γ secretion in the colon, the kidneys, and even in the serum to basal concentrations on day 6 p.i. In conclusion, carvacrol alone and its combination with deferoxamine, deoxycholate, and 2-fucosyl-lactose constitute promising antibiotics-independent treatment options to fight acute campylobacteriosis.

## 1 Introduction

Campylobacteriosis is an acute infectious enteritis, primarily caused by foodborne *Campylobacter* species transmitted through contaminated chicken and turkey products, with *Campylobacter jejuni* being the most common pathogen (Price et al., 1979; Walker et al., 1986; Moore et al., 2005; Young et al., 2007; Kaakoush et al., 2015; Wagenaar et al., 2023). Given that the intestinal tract of poultry represents the major pathogen reservoir, the global surge in consumption of poultry products has contributed significantly to the continuous rise of human *C. jejuni* infections. As a result, campylobacteriosis has emerged as a critical global health problem with a high socioeconomic burden (Kaakoush et al., 2015; WHO, 2020; European Food Safety Authority, European Centre for Disease Prevention and Control, 2022). The asymptomatic colonization of avian intestinal tracts by the highly virulent enteropathogen has far-reaching implications for their transmission to humans. The commensal lifestyle in birds is the major reason for the tolerance of *C. jejuni* contaminations in poultry production chains and favors its adaptation to the intestines (Moore et al., 2005; Young et al., 2007; Kaakoush et al., 2015). The lack of disease symptoms in birds can be attributed to the avian innate immune system’s non-responsiveness to the pathogen. Additionally, birds display a resistance to lipopolysaccharide (LPS), a cell wall structure of Gram-negative bacteria, including *Campylobacter* species. *C. jejuni* produces a truncated version of LPS known as lipo-oligosaccharide (LOS), which further complicates pathogen recognition. Previous studies suggest that these variations in pathogen recognition may arise from species-specific differences in how *Campylobacter* LOS interacts with the specific Toll-like receptor (TLR)-4 (de Zoete et al., 2010).

In contrast to the avian hosts, humans exhibit heightened sensitivity to TLR-4 ligands such as LPS and LOS of Gram-negative bacteria. Consequently, when *C. jejuni* infects the human intestinal tract, LOS triggers host immune responses and induces hyperactivation of the innate and adaptive immune system via TLR-4 and mammalian target of rapamycin (mTOR) signaling (Sun et al., 2012; Callahan et al., 2021). This cascade results in damage to intestinal cells such as apoptosis and dissolution of the tight junctions (Lobo de Sá et al., 2021). Subsequently, patients experience acute enterocolitis characterized by bloody diarrhea, intestinal cellular apoptosis, compromised barrier function, malabsorption, and ultimately, severe tissue destruction (Sun et al., 2013; Lobo de Sá et al., 2021).

Although the majority of human campylobacteriosis cases resolve without residues within 1 to 2 weeks post-infection (p.i.), the potential to develop post-infectious autoimmune diseases, including Guillain-Barré syndrome, reactive arthritis, and intestinal diseases, including chronic inflammatory bowel diseases and irritable bowel syndrome remains (Nachamkin et al., 1998; Mortensen et al., 2009; Omarova et al., 2023). Importantly, the risk for these secondary complications correlates with the severity of the initial enteritis (Mortensen et al., 2009). Therefore, severe and invasive *C. jejuni* induced enterocolitis presenting with bloody diarrhea may require antibiotic therapy, particularly in immunocompromised and multimorbid individuals (Manfredi et al., 1999; Acheson and Allos, 2001). However, the increasing prevalence of antibiotic resistance in *Campylobacter* strains, including resistance to commonly used quinolones and macrolides, restricts the efficacy of antimicrobial therapy for human campylobacteriosis (Mouftah et al., 2021). Hence, there is an urgent need to develop novel antibiotic-independent strategies to combat and/or prevent *C. jejuni* infections, aligning with the One-Health approach (Zhang et al., 2023).

To promote global health and address pressing issues like antibiotic resistance and foodborne infections, the One Health concept seeks to balance and improve the health of humans, animals, and ecosystems (Anholt and Barkema, 2021). According to the One Health approach, multi-sectoral (i.e., humans, animals, and the associated environments) and inter-disciplinary strategies (involving farming, human and veterinary medicine for instance) are crucial to combat antibiotic resistance and their collateral damages (Robinson et al., 2016). Therefore, it is important to develop pharmaceutical intervention strategies to treat human campylobacteriosis with antibiotic-independent compounds. Additionally, it is important to identify alternative measures to control antibiotic resistance in both human hosts and animal reservoirs. These actions would enhance safety aspects along the food chains and reduce morbidities in humans caused by transmitted pathogens.

In fact, current research efforts have shifted towards identifying natural compounds with both anti-microbial and anti-inflammatory properties that do not trigger resistance. These compounds hold promise for future therapeutic and preventive applications in the context of enteropathogenic infections, including those caused by Campylobacter species (Kreling et al., 2020).

Carvacrol, a phenolic monoterpenoid and main component in essential oils derived from oregano (*Origanum vulgare*) and thyme (*Thymus vulgaris*) (Gholami-Ahangaran et al., 2022), is commonly used for flavoring and food-preservation within the food industry. Furthermore, it is known for its anti-microbial, −oxidant, −diabetic, −inflammatory, and anti-carcinogenic properties, making this compound a promising candidate in the treatment of several human morbidities (Sharifi-Rad et al., 2018; Mączka et al., 2023). The anti-bacterial effects of carvacrol against food-borne pathogens, such as Gram-positive *Bacillus cereus* or Gram-negative bacteria like *Escherichia coli*, *Salmonella* species, and *C. jejuni* have already been reported in the past (Ultee et al., 2002; Obaidat and Frank, 2009; van Alphen et al., 2012; Mączka et al., 2023). Additionally, our previous pre-clinical intervention studies assessed the anti-inflammatory and immune-modulatory properties of singular carvacrol application during murine campylobacteriosis (Mousavi et al., 2020; Foote et al., 2023).

Deferoxamine B (deferoxamine, trade name Desferal^®^) is an iron chelator and natural siderophore produced by microorganisms such as *Streptomyces pilosus*. This compound exhibits anti-microbial, anti-inflammatory, and cell-protective properties (Jurado, 1997; Bellotti and Remelli, 2021). Given that iron is essential for the survival of most pathogens, iron binding by deferoxamine can reduce the risk of infections (Jurado, 1997). Additionally, several studies revealed synergistic effects between deferoxamine and anti-microbial agents such as gentamicin, metronidazole, and hydrogen peroxide (Van Asbeck et al., 1983; van Asbeck et al., 1983; Moon et al., 2011). Previously we reported that the oral application of deferoxamine significantly improved the clinical outcome of *C. jejuni* infected mice, which was accompanied by dampened inflammatory responses such as less pronounced colonic epithelial cell apoptosis as compared to placebo-treated infected mice (Bereswill et al., 2022).

The secondary bile acid deoxycholic acid has been shown to exhibit bactericidal effects against several bacteria, including *Staphylococcus aureus* (Zhao et al., 2023) and *Helicobacter pylori* (Itoh et al., 1999). Previous studies revealed that *C. jejuni* alter the global gene transcription in response to the bile salt deoxycholate (Gourley et al., 2017; Negretti et al., 2017). Remarkably, deoxycholic acid effectively reduced *C. jejuni* growth *in vitro* (Negretti et al., 2017) and its colonization capacity in the intestinal tract of broiler chickens (Alrubaye et al., 2019). Furthermore, deoxycholic acid possesses immune-modulatory activities such as suppressing LPS-induced expression of pro-inflammatory cytokines such as interleukin (IL)-1, IL-6, and tumor necrosis factor-alpha (TNF-α) in murine bone marrow-derived dendritic cells (Hu et al., 2021; Su et al., 2023).

Human breast milk is the main source of energy in the early stage of life. However, the lack of enzymes responsible for the release of monosaccharides makes the human milk oligosaccharides (HMOs) indigestible by the newborn (Engfer et al., 2000; Sela and Mills, 2010). Interestingly, distinct bacteria such as *Bifidobacterium* and *Lactobacillus* species use HMOs as nutrients, which indicates the prebiotic properties of HMOs by promoting intestinal colonization by a “healthy” gut microbiota (Davis et al., 2016; Garrido et al., 2016). Additionally, 2-fucosyl-lactose, one of the most abundant oligosaccharides in human breast milk is known for its immune-modulatory effects and has been shown to prevent the binding of pathogens such as *Pseudomonas aeruginosa, E. coli,* and *C. jejuni* to the host epithelial cells (Newburg, 2005; Facinelli et al., 2019).

So far, research on *C. jejuni*-host interactions has been limited by standardized *in vivo* models. The commensal gut microbiome of conventional laboratory mice exerts a strong physiological colonization resistance, which prevents infections with enteropathogens like *C. jejuni* (Mullineaux-Sanders et al., 2018; Herzog et al., 2023). Therefore, an antibiotic-pretreatment regimen of mice has been established to generate proper enteropathogenic infection models. After depleting the murine microbiome, a stable colonization of *C. jejuni* through oral gavage can be assured (Bereswill et al., 2011). However, even though *C. jejuni* colonizes in secondary abiotic wildtype mice, characteristic clinical symptoms of acute human campylobacteriosis remain absent (Bereswill et al., 2011). This is due to the resistance against TLR-4 ligands like LPS and LOS, which is 10,000 times stronger in rodents as compared to humans (Mortensen et al., 2009). To overcome this challenge, the murine *il10* gene has been knocked out to make the animals more susceptible to *C. jejuni* LOS (Mansfield et al., 2007). In consequence, secondary abiotic IL-10^−/−^ mice can be colonized by the enteropathogen and also display *C. jejuni*-induced acute enterocolitis within a week post infection (p.i.) mimicking key features of severe and invasive human campylobacteriosis (Bereswill et al., 2011; Haag et al., 2012). Our previous preclinical placebo-controlled intervention studies using this acute *C. jejuni* infection and inflammation model provided evidence for anti-pathogenic and immune-modulatory properties of several natural compounds such as carvacrol (Mousavi et al., 2020), deferoxamine (Bereswill et al., 2022), and 2-fucosyl-lactose (Mousavi et al., 2023).

Previous studies also revealed that the host-specific intestinal microbiota impacts the host’s susceptibility to and resistance against distinct enteropathogens including *C. jejuni* (Bereswill et al., 2011), *Campylobacter coli* (Heimesaat et al., 2020), and *Salmonella enterica* (Chung et al., 2012). In order to elucidate the triangular relationship between enteropathogens on one side and the murine immunity and human gut microbiota on the other, the host side, we generated a human gut microbiota associated (hma) mouse model for *C. jejuni* induced inflammation. Therefore, secondary abiotic IL-10^−/−^ mice were subjected to oral transplantation of a fecal microbiota from healthy human donors (Foote et al., 2023; Shayya et al., 2023). Following oral *C. jejuni* infection, human gut microbiota associated IL-10^−/−^ mice were shown not only to carry the pathogen in their intestines at high loads but also to present with key symptoms of acute campylobacteriosis like secondary abiotic mice do (Shayya et al., 2023).

This preclinical placebo-controlled intervention study assesses carvacrol, deferoxamine, deoxycholic acid, and 2′-fucosyl-lactose alone and as a combination in hma IL-10^−/−^ mice with acute *C. jejuni* induced enterocolitis. The following parameters will be addressed: (i) human gut microbiota compositions in recipient mice following engraftment; (ii) gastrointestinal pathogens burdens; (iii) clinical outcome; (iv) intestinal, (v) extra-intestinal, and also (vi) the systemic inflammatory immune responses upon oral infection.

## 2 Methods

### 2.1 Ethics

All animal procedures were performed in accordance with protocols approved by the local commission for animal experiments (“Landesamt für Gesundheit und Soziales,” LaGeSo, Berlin; registration number G0104/19). Clinical conditions of the mice were assessed daily following the European animal welfare guidelines (2010/63/EU).

### 2.2 Secondary abiotic IL-10^−/−^ mice

IL-10^−/−^ C57BL/6j mice were obtained from the Forschungsinstitute für Experimentelle Medizin of the Charité – Universitätsmedizin Berlin, Germany. Animals were bred and kept under a specified pathogen-free environment, housed in autoclaved cages with filter tops within an experimental semi-barrier, and given free access to standard chow (food pellets: ssniff R/M-H, V1534-300, Sniff, Soest, Germany) as well as autoclaved tap water. In all experiments, age- and sex-matched littermates were used. Mice were 3-week-old when subjected to an 8-week antibiotic treatment consisting of ampicillin plus sulbactam (2 g/L plus 1 g/L, respectively; Dr. Friedrich Eberth Arzneimittel, Ursensollen, Germany) *ad libitum*. To avoid cross-contamination, secondary abiotic mice were handled under aseptic conditions throughout the experiment. After eradication of the murine microbiome as described previously (Heimesaat et al., 2022), and 2 days prior to human fecal microbiota transplantation (FMT), the antibiotic treatment was substituted with autoclaved tap water to ensure proper washout.

### 2.3 Human fecal microbiota transplantation

**Figure 1:**
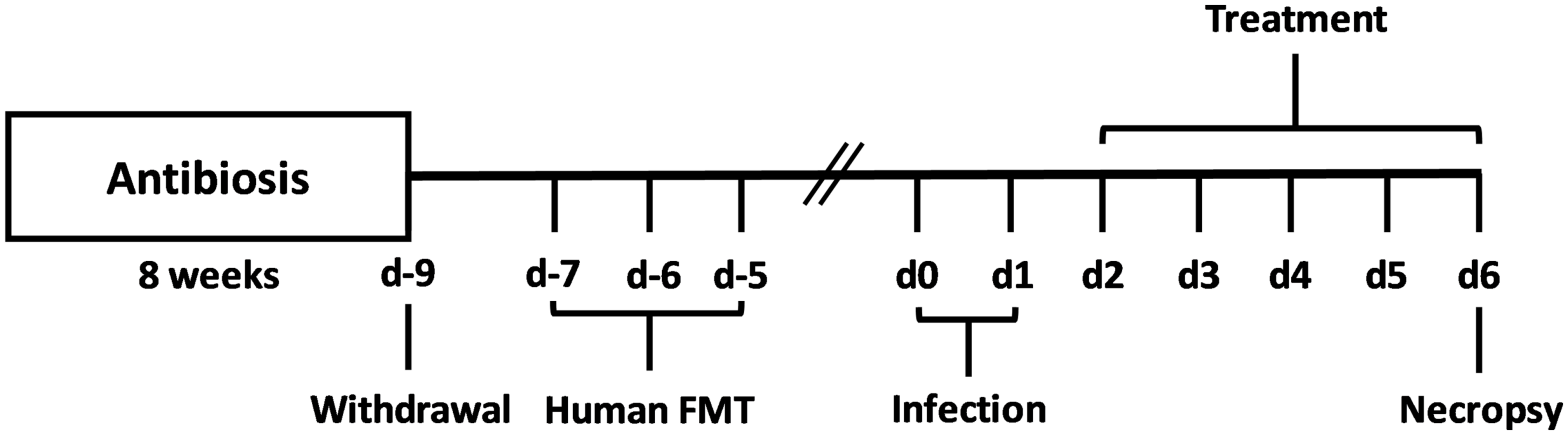
Experimental set-up. For gut microbiota depletion, conventionally raised IL-10^−/−^ mice were subjected to an 8-week pretreatment with ampicillin plus sulbactam. Two days before oral human fecal microbiota transplantation (FMT) of secondary abiotic IL-10^−/−^ mice on day (d)-7, d-6, and d-5, the antibiotic compounds were replaced by sterile tap water. On d0 and d1, human microbiota associated (hma) mice were perorally infected with *C. jejuni* strain 81–176, and treated with synthetic carvacrol, deferoxamine, deoxycholic acid, and 2-fucosyl-lactose alone, with a combination of all four compounds or with placebo (via the drinking water) from d2 until necropsy on d6 post-infection.

Human fecal samples were collected from five healthy donors free of viruses, parasites, as well as enteropathogenic bacteria and stored at −80°C. After thawing and resuspending in sterile phosphate buffered saline (PBS, Thermo Fisher Scientific, Waltham, MA, United States), the samples were pooled to ensure at least 0.3 mL gavage volume per mouse. The microbiota composition of the pooled FMT sample can be found in Supplementary Figure S1. One week before the first *C. jejuni* infection (respectively on days 0 and 1), secondary abiotic mice were introduced to the complex human FMT on 3 days (respectively on days −7, −6 and −5) (Figure 1) as described previously (Shayya et al., 2023).

### 2.4 *Campylobacter jejuni* cultures and infections

Viable cells of *C. jejuni* strain 81–176 were thawed from −80°C and grown on *Campylobacter*-selective Karmali agar plates (Oxoid, Wesel, Germany) under microaerophilic conditions (CampyGen gas packs, Oxoid, Wesel, Germany) at 37°C (Bereswill et al., 2011). After 48 h, plates were harvested and bacterial cells were resuspended in sterile PBS to achieve an inoculum of 10^9^ colony forming units (CFU). Then, 0.3 mL per mouse were gavaged on days 0 and 1 as shown previously (Bereswill et al., 2011).

### 2.5 Course of treatment

Treatment through oral administration via drinking water of carvacrol, deferoxamine, deoxycholic acid (all from Sigma-Aldrich, Munich, Germany), 2′-fucosyl-lactose (Chr. Hansen HMO GmbH, Rheinbreitbach, Germany), and the quadruple combination commenced 2 days following the onset of *C. jejuni* infection. Dosages were calculated considering an average body weight of approximately 25 g per mouse and an estimated drinking volume of 5 mL per day. All compounds were dissolved in autoclaved tap water. The daily doses and their previously determined minimal inhibitory concentrations (MICs) are illustrated in Table 1. To enhance the water solubility of carvacrol, 50 mg of the compound were dissolved in 250 μL Tween^®^ 80 (Sigma-Aldrich, Munich, Germany). The placebo group received vehicle dissolved in autoclaved tap water.

**Table 1:** Treatment regimens and concentrations of applied substances.

Treatment	Daily Dose (mg/kg)	Drinking Solution (mg/L)	MIC^a^ (mg/L)
Placebo	–	–	–
Carvacrol	100	500	150
Deferoxamine	100	500	8
Deoxycholic acid	50	250	>256
2-Fucosyl-lactose	480	2,400	>32,768
Combination	730	3,650	114

a MIC: minimal inhibitory concentration.

### 2.6 *Campylobacter jejuni* loads in the gastrointestinal tract

Intraluminal samples from defined parts of the gastrointestinal tract (stomach, duodenum, ileum, and colon) were taken upon necropsy of the mice. The samples were homogenized in PBS, serial diluted and streaked on Karmali agar plates (Oxoid, Wesel, Germany). After 48 h of incubation at 37°C under microaerophilic conditions, *C. jejuni* was quantified by counting CFU (Bereswill et al., 2011). The detection limit of viable bacterial cells was 100 CFU per g intestinal sample.

### 2.7 Microbiota analysis

Fecal samples of hma mice were collected before (i.e., day 0) and 6 days after *C. jejuni* infection for microbiota composition analysis in comparison to the original fecal donor suspensions (Heimesaat et al., 2006). Samples were homogenized in sterile PBS, diluted in series and incubated on solid media under aerobic, microaerobic, and anaerobic conditions for 48 h. Total bacterial loads and bacterial species were identified according to their colony morphology, Gram-staining, and biochemical analysis. In addition, genomic DNA extraction and real-time polymerase chain reaction (PCR) of the fecal samples have been performed culture-independently; this is to assess the abundance of fastidious and non-cultivable bacteria from the human microbiome quantitatively. 16S variable regions were targeted using species-, genera- or group-specific 16S rRNA primers (Tib MolBiol, Berlin, Germany) (Heimesaat et al., 2006). Results are illustrated as 16S rRNA gene copies per ng DNA.

### 2.8 Clinical outcomes

Health outcome of mice was monitored before and for 6 days after *C. jejuni* infection. Total clinical score (maximum 12 points) constituted of clinical aspect (i.e., wasting symptoms; 0: normal; 1: ruffled fur; 2: less locomotion; 3: isolation; 4: severely compromised locomotion, pre-final aspect), fecal blood (0: none; 2: microscopic detection using the Guajac method (Haemoccult, Beckman Coulter/PCD, Germany); 4: visible blood spots), and stool appearance (0: normal/firm; 2: pasty; 4: liquid), as stated previously (Heimesaat et al., 2014).

### 2.9 Dissection and sampling

Six days p.i., mice were sacrificed by carbon dioxide asphyxiation and dissected in an aseptic environment. *Ex vivo* tissue samples from liver, kidneys, mesenteric lymph nodes (MLN), colon, in addition to luminal samples (from stomach, duodenum, ileum, and colon) were collected for microbiological, immunological, and immunohistopathological analyses. Heart blood was used for cytokine measurements.

### 2.10 Histopathological changes

Colonic tissue samples were fixed in 5% formalin and embedded in paraffin. 5-μm-sections were then stained with hematoxylin and eosin (H&E) to evaluate histopathological changes. The colonic mucosa was assessed under light microscopy (100 × magnification) using the following scoring scheme (Erben et al., 2014): Score 0, normal/no inflammatory cell infiltrates in epithelium. Score 1, minimal inflammatory cell infiltrates in mucosa but still intact epithelium. Score 2, mild inflammatory cell infiltrates in mucosa and submucosa with mild hyperplasia and mild goblet cell loss. Score 3, moderate inflammatory cell infiltrates in mucosa with moderate goblet cell loss. Score 4, extensive inflammatory cell infiltration into mucosa and submucosa with marked goblet cell loss, multiple crypt abscesses, and crypt loss.

### 2.11 *In situ* immunohistochemistry

5-μm-sections of colonic tissue samples were fixed in 5% formalin and embedded in paraffin as previously described (Heimesaat et al., 2018; Foote et al., 2023). Primary antibodies against cleaved caspase-3 (Asp175, Cell Signaling, Beverly, MA, United States, 1:200), MPO7 (No. A0398, Dako, Glostrup, Denmark, 1:500), CD3 (no. N1580, Dako, 1:10), and FOXP3 (clone FJK-165, no. 14-5773, eBioscience, 1:100) were used to count apoptotic epithelial cells, neutrophils, T lymphocytes, and regulatory T cells under light microscopy. The mean number of detected cells in each blinded sample was determined within at least six high power fields (HPF, 0.287 mm^2^, 400 × magnification).

### 2.12 Mouse inflammation cytometric bead assay

*Ex vivo* tissue samples from colon, liver (both approximately 1 cm^3^), kidney (one half after the longitudinal cut) were collected and washed in sterile PBS. Samples were incubated at 37°C for 18 h in 24-flat-bottom well culture plates (Thermo Fisher Scientific, Waltham, MA, United States) containing 500 μL serum-free RPMI 1640 medium (Thermo Fisher Scientific, Waltham, MA, United States), penicillin (100 μg/mL; Biochrom, Berlin, Germany) and streptomycin (100 μg/mL; Biochrom, Berlin, Germany). The culture supernatants and serum samples were then tested for interferon-gamma (IFN-γ) and IL-6 by applying the Mouse Inflammation Cytometric Bead Assay (BD Biosciences, Germany) in a BD FACS Canto II flow cytometer (BD Biosciences).

### 2.13 Statistical analysis and reproducibility

Results from animal experiments are representative of three independent repetitions. Statistical analysis was performed using GraphPad Prism (version 9; San Diego, CA, United States). The Anderson-Darling test was used to normalize data sets. The Student’s *t*-test and Mann–Whitney test were applied for pairwise comparisons of normally and not normally distributed data. Multiple comparisons were performed using the one-way ANOVA with Tukey post-correction (for normally distributed data) and Kruskal-Wallis test with Dunn’s post-correction (for not normally distributed data). *p* values of less than 0.05 were considered significant. Outliers were identified by the Grubb’s test.

## 3 Results

### 3.1 Fecal microbiota composition in hma IL-10^−/−^ mice immediately before *Campylobacter jejuni* infection

**Figure 2:**
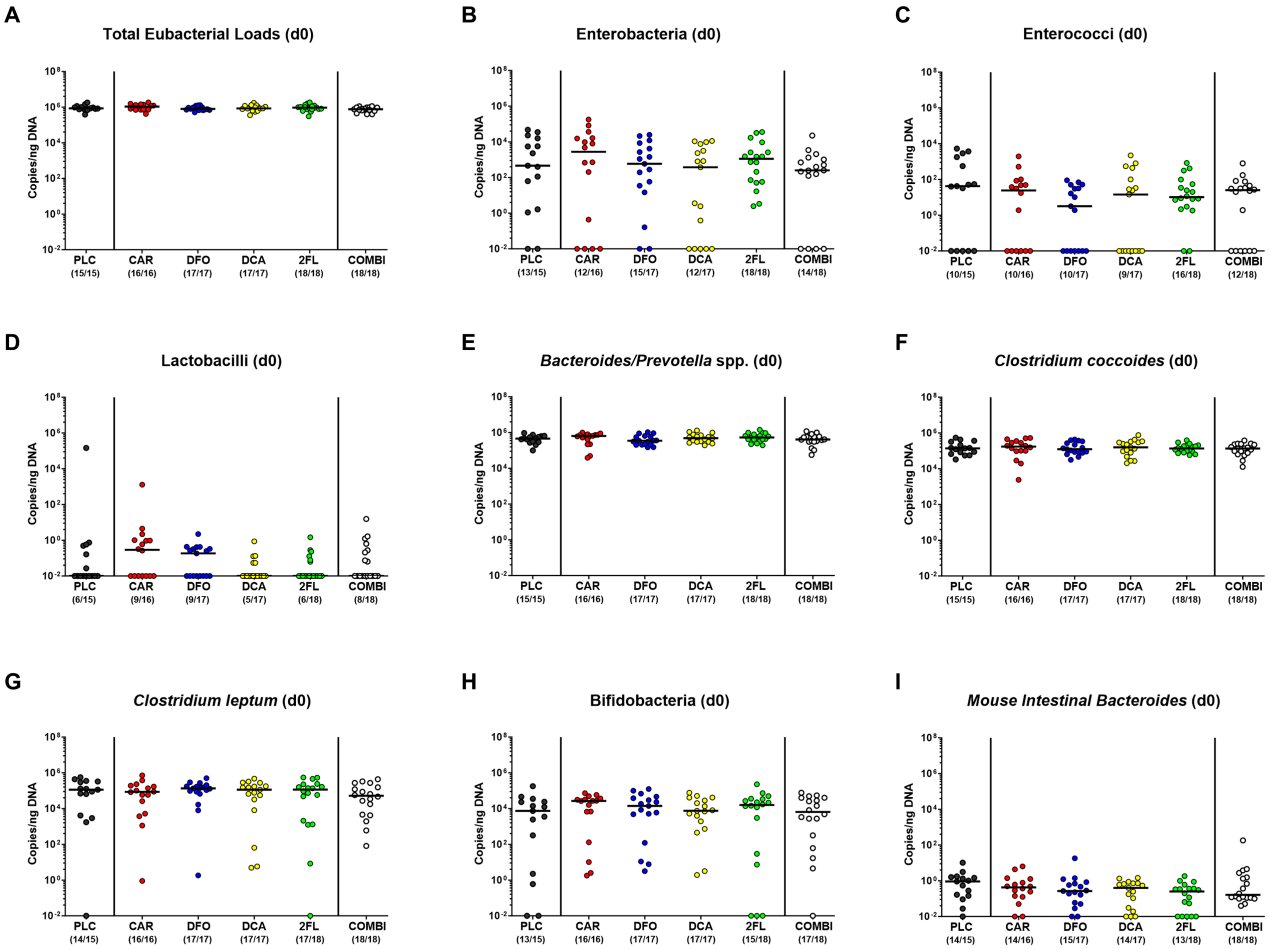
Culture-independent analysis of fecal microbiota composition in human microbiota associated IL-10^−/−^ mice immediately before *C. jejuni* infection. A week before *C. jejuni* infection, secondary abiotic IL-10^−/−^ mice (that had been generated by antibiotic pretreatment) were subjected to human fecal microbiota transplantation on three consecutive days by oral gavage [i.e., on day (d)-7, d-6, and d-5]. Immediately before *C. jejuni* infection on d0, the fecal microbiota composition was surveyed in mice from the prospective treatment cohorts (PLC, placebo; CAR, carvacrol; DFO, deferoxamine; DCA, deoxycholic acid; 2FL, 2-fucosyl-lactose; COMBI, a combination of all four compounds) by culture-independent, 16S rRNA real-time PCR (see methods) assessing the **(A)** total eubacterial loads, **(B)** enterobacteria, **(C)** enterococci, **(D)** lactobacilli, **(E)** *Bacteroides/Prevotella* species (spp.), **(F)** *Clostridium coccoides* group, **(G)** *Clostridium leptum* group, **(H)** bifidobacteria, and **(I)** *Mouse Intestinal Bacteroides* (expressed as copies per ng DNA). Medians (black bars) and numbers of mice with bacteria-positive detection out of the total number of analyzed animals (in parentheses) are indicated.

We first assessed whether the human fecal microbiota transplants had comparably engrafted in the intestinal tract of secondary abiotic mice within 1-week after triple human FMTs. To address this, we performed quantitative gut microbiota analyses immediately before *C. jejuni* infection. Both, cultural and culture-independent (i.e., molecular) analyses proved that the fecal loads of the tested intestinal bacterial groups, genera, and species were comparably high in the six prospective treatment cohorts of the hma IL-10^−/−^ mice pointing towards similar engraftment of the human fecal transplants in the murine hosts (Supplementary Figure S2; Figure 2).

### 3.2 Gastrointestinal *Campylobacter jejuni* loads following oral infection and treatment of hma IL-10^−/−^ mice with carvacrol, deferoxamine, deoxycholic acid, and 2-fucosyl-lactose alone or in combination

**Figure 3:**
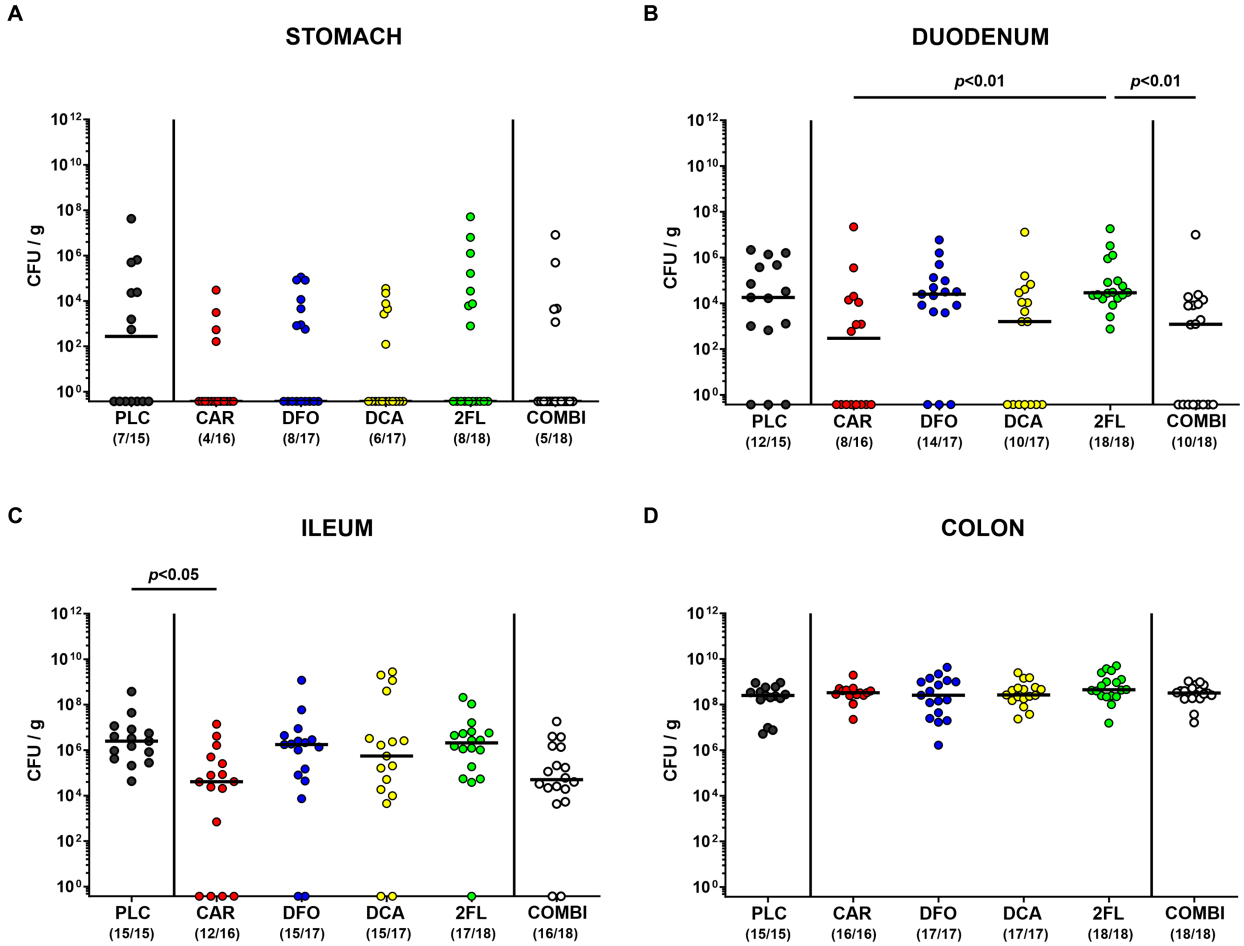
Establishment of *C. jejuni* in the gastrointestinal tract of infected hma IL-10^−/−^ mice. Hma IL-10^−/−^ mice were orally infected with *C. jejuni* strain 81–176 on days 0 and 1. From day 2 until day 6 post-infection, mice were treated with synthetic carvacrol (CAR), deferoxamine (DFO), deoxycholic acid (DCA), 2-fucosyl-lactose (2FL), a combination of all four compounds (COMBI) or placebo (PLC) via the drinking water. Upon sacrifice on d6 post-infection, *C. jejuni* were quantitated in luminal samples taken from the **(A)** stomach, **(B)** duodenum, **(C)** ileum, and **(D)** colon by culture and expressed as colony-forming units per gram (CFU/g). Individual data pooled from three experiments, medians (black bars), the numbers of culture-positive mice out of the total number of analyzed animals (in parentheses), and the significance levels (*p* values) determined by the Kruskal-Wallis test and Dunn’s post-correction are shown.

Following the start of oral treatment in *C. jejuni* infected hma IL-10^−/−^ mice with carvacrol, deferoxamine, deoxycholic acid, and 2-fucosyl-lactose alone or all four in combination on day 2 p.i., we surveyed the *C. jejuni* pathogen loads in fecal samples over time. Analyses by culture revealed median fecal *C. jejuni* counts of approximately 10^9^ viable bacteria per gram that did not differ between the six treatment cohorts on days 2, 3, 4, and 5 p.i. [not significant (n.s.); Supplementary Figure S3]. Furthermore, we enumerated viable *C. jejuni* bacteria in luminal samples taken from defined gastrointestinal parts upon necropsy. Whereas the pathogen counts in the stomach and colon did not differ between the respective groups (n.s.; Figures 3A,D), approximately two orders of magnitude lower pathogen numbers could be cultured from the ileum lumen of carvacrol as compared to placebo treated mice on day 6 p.i. (*p* < 0.05; Figure 3C). Furthermore, the carvacrol and combination groups exhibited lower *C. jejuni* burdens in their duodenum as compared to 2-fucosyl-lactose treated mice (*p* < 0.01; Figure 3B). Therefore, all treatment regimens, whether applied individually or in combination did not affect *C. jejuni* loads in the colon. It is notable that only the carvacrol treatment lowered pathogen loads in the ileal tracts of infected hma IL-10^−/−^ mice.

### 3.3 Clinical outcomes of hma IL-10^−/−^ mice following *Campylobacter jejuni* infection and treatment with carvacrol, deferoxamine, deoxycholic acid, and 2-fucosyl-lactose alone or in combination

**Figure 4:**
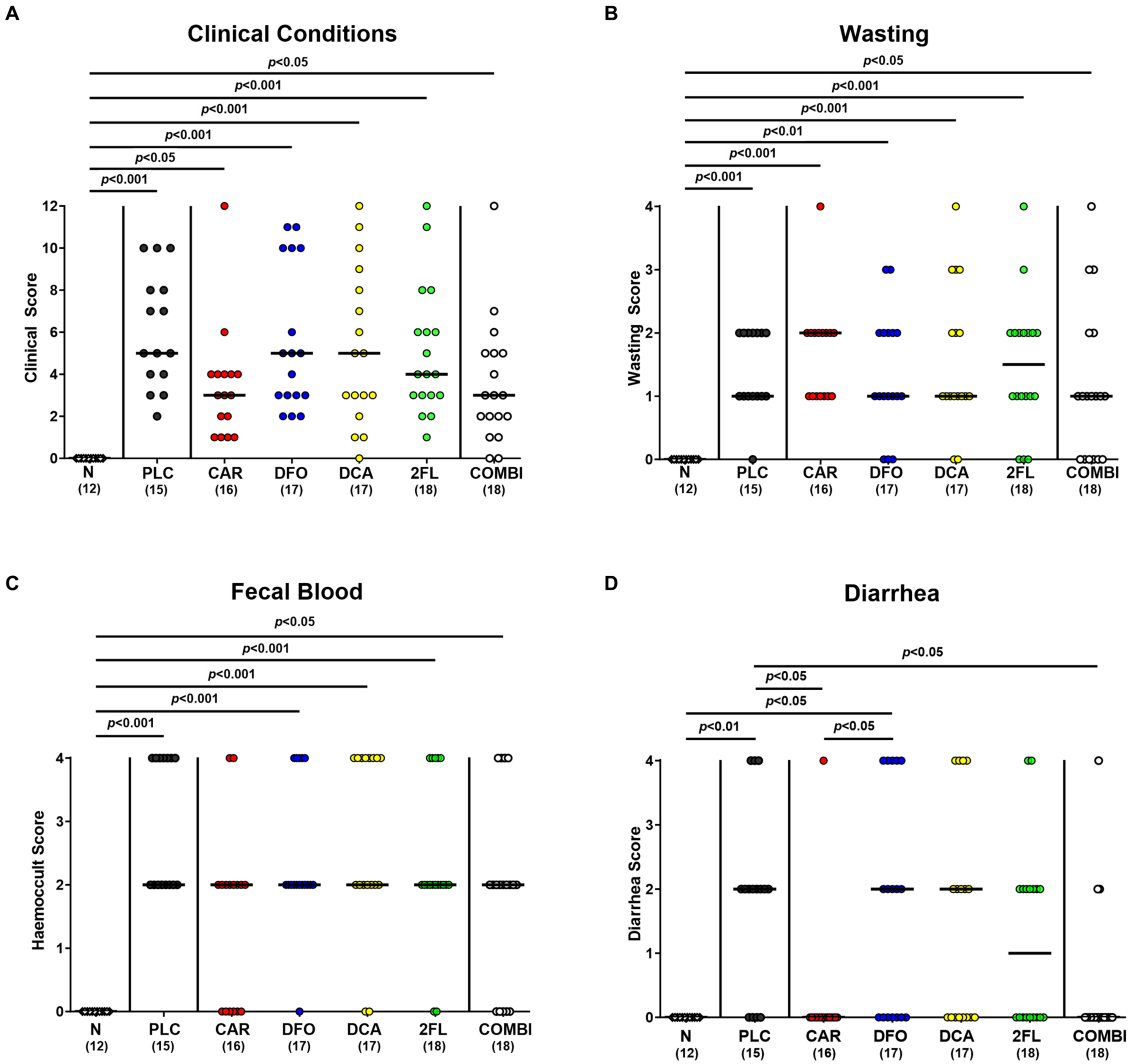
Clinical outcomes in hma IL-10^−/−^ mice following *C. jejuni* infection and treatment with carvacrol, deferoxamine, deoxycholic acid, and 2-fucosyl-lactose alone or all in combination. *C. jejuni* infected hma IL-10^−/−^ mice were perorally treated with synthetic carvacrol (CAR), deferoxamine (DFO), deoxycholic acid (DCA), 2-fucosyl-lactose (2FL), a combination of all four compounds (COMBI) or placebo (PLC) from day 2 until day 6 post-infection. At the end of the treatment (i.e., day 6 post-infection), the clinical outcomes were quantitated with clinical scores assessing the **(A)** overall clinical conditions and specifically, **(B)** wasting symptoms, **(C)** diarrhea, and **(D)** fecal blood. Naive (N) hma IL-10^−/−^ mice served as non-infected and untreated controls. Individual data pooled from three experiments, the medians (black bars), the numbers of included mice (in parentheses), and the significance levels (*p* values) determined by the Kruskal-Wallis test and Dunn’s post-correction are shown.

We further surveyed the effect of the applied treatment regimens on *C. jejuni* induced disease with campylobacteriosis scores (assessing wasting symptoms plus diarrhea plus fecal blood) over time p.i. As early as 24 h after initiation of the treatments (i.e., on day 3 p.i.), increased clinical scores were determined in the deferoxamine, deoxycholic acid, 2-fucosyl-lactose, and placebo treated groups (*p* < 0.01–0.001 versus naive; Supplementary Figure S4A), whereas carvacrol and combination treated hma mice displayed basal values (n.s. versus naive; Supplementary Figure S4A). This also held true for basal scores in mice from the combination group on days 4 and 5 p.i. (n.s. versus naive; Supplementary Figures S4B,C). Immediately before necropsy on day 6 p.i., mice from all infected cohorts exhibited similarly increased overall campylobacteriosis scores (*p* < 0.05–0.001 versus naive; Figure 4A), but with a trend towards lower median scores in carvacrol and combination treated mice versus placebo counterparts (n.s. due to relatively high standard deviations; Figure 4A). When specifically quantitating wasting symptoms on day 6 p.i., no differences in wasting scores could be observed between treated mice (*p* < 0.05–0.001 versus naive; Figure 4B), whereas the scores for fecal blood were increased in all cohorts except the carvacrol treatment group (n.s. versus naive; Figure 4C). Remarkably, carvacrol and combination treated mice suffered from less severe diarrheal symptoms as indicated by lower diarrhea scores if compared to placebo control mice on day 6 p.i. (*p* < 0.05; Figure 4D). Of note, mice from the carvacrol, deoxycholic acid, 2-fucosyl-lactose, and combination cohorts exhibited basal diarrheal scores at the end of the experiment (n.s. versus naive; Figure 4D). Hence, carvacrol and combination treatment alleviated diarrheal symptoms in *C. jejuni* infected hma mice.

### 3.4 Macroscopic and microscopic inflammatory changes in hma IL-10^−/−^ mice following *Campylobacter jejuni* infection and treatment with carvacrol, deferoxamine, deoxycholic acid, and 2-fucosyl-lactose alone or in combination

**Figure 5:**
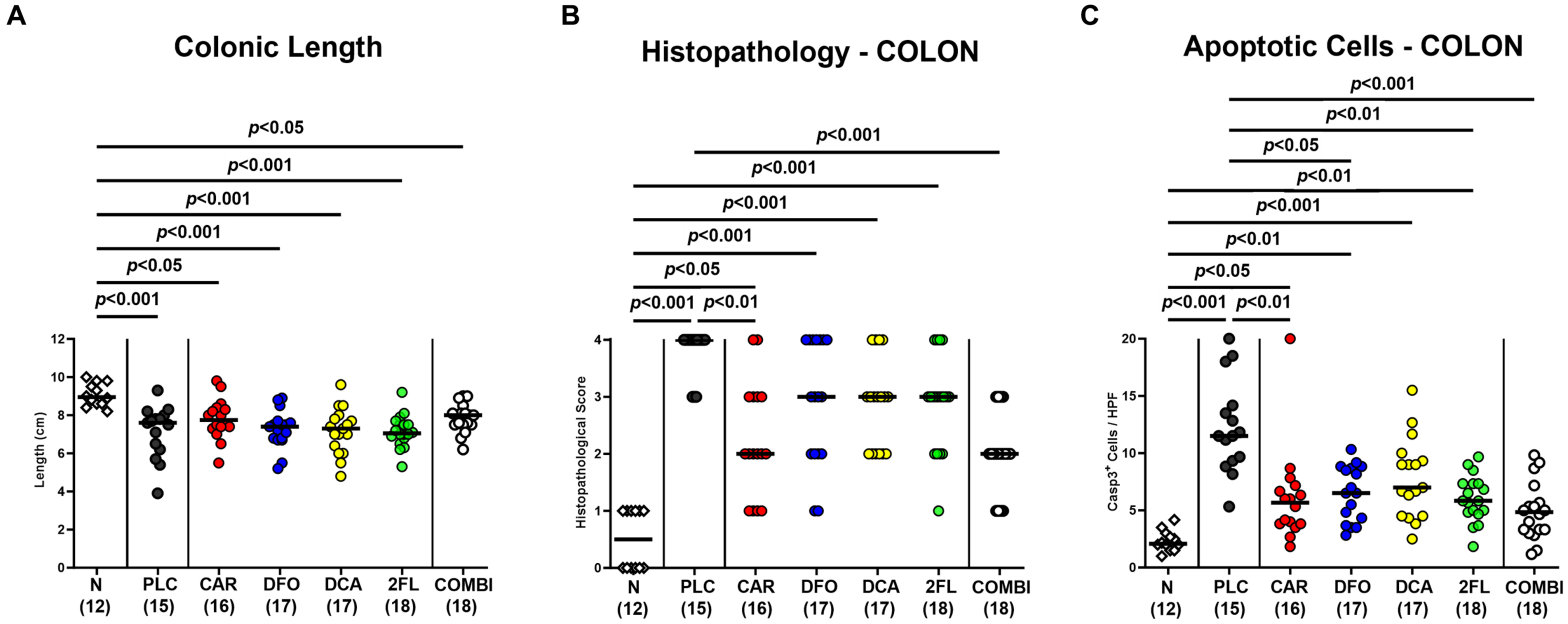
Macroscopic and microscopic inflammatory changes in hma IL-10^−/−^ mice following *C. jejuni* infection and treatment with carvacrol, deferoxamine, deoxycholic acid, and 2-fucosyl-lactose alone or all in combination. *C. jejuni* infected hma IL-10^−/−^ mice were perorally treated with synthetic carvacrol (CAR), deferoxamine (DFO), deoxycholic acid (DCA), 2-fucosyl-lactose (2FL), a combination of the four compounds (COMBI) or placebo (PLC) via the drinking water from day 2 until day 6 post-infection. On day 6, the **(A)** colonic lengths were measured, the **(B)** histopathological changes scored in hematoxylin and eosin-stained colonic paraffin sections, and the **(C)** numbers of apoptotic colonic epithelial cells determined in colonic paraffin sections stained with an antibody against cleaved caspase-3 (average numbers out of six representative high-power fields (HPF, 400 × magnification) per mouse). Naive (N) hma IL-10^−/−^ mice served as non-infected and untreated controls. Individual data pooled from three experiments, the medians (black bars), the numbers of included mice (in parentheses), and the significance levels (*p* values) determined by the one-way ANOVA test with Tukey post-correction **(A)** and by the Kruskal-Wallis test and Dunn’s post-correction **(B,C)** are shown.

Next, we assessed changes in colonic lengths, as enterocolitis is known to result in significantly shorter intestines due to inflammation (Heimesaat et al., 2006; Bereswill et al., 2011). Irrespective of the treatment, infected mice displayed shorter colons as compared to uninfected control animals (*p* < 0.05–0.001; Figure 5A). Furthermore, we quantitated the *C. jejuni* induced histopathological changes in the colon and determined lower histopathological scores in carvacrol and combination treated mice as compared to placebo counterparts on day 6 p.i., indicative for less severe pathogen-induced microscopic disease (*p* < 0.01 and *p* < 0.001, respectively; Figure 5B). In addition, the numbers of apoptotic colonic epithelial cells were lower in infected mice from the carvacrol, deferoxamine, 2-fucosyl-lactose, and combination cohorts versus placebo (*p* < 0.05–0.001; Figure 5C). The combination treatment dampened both, histopathological and apoptotic cell responses in the colon of *C. jejuni* infected mice to basal levels (n.s. versus naive; Figures 5B,C). Hence, carvacrol and combination treatment of hma IL-10^−/−^ mice resulted in diminished microscopic (i.e., histopathological and apoptotic) sequelae of *C. jejuni* infection.

### 3.5 Immune cell subsets in the colon of hma IL-10^−/−^ mice following *Campylobacter jejuni* infection and treatment with carvacrol, deferoxamine, deoxycholic acid, and 2-fucosyl-lactose alone or in combination

**Figure 6:**
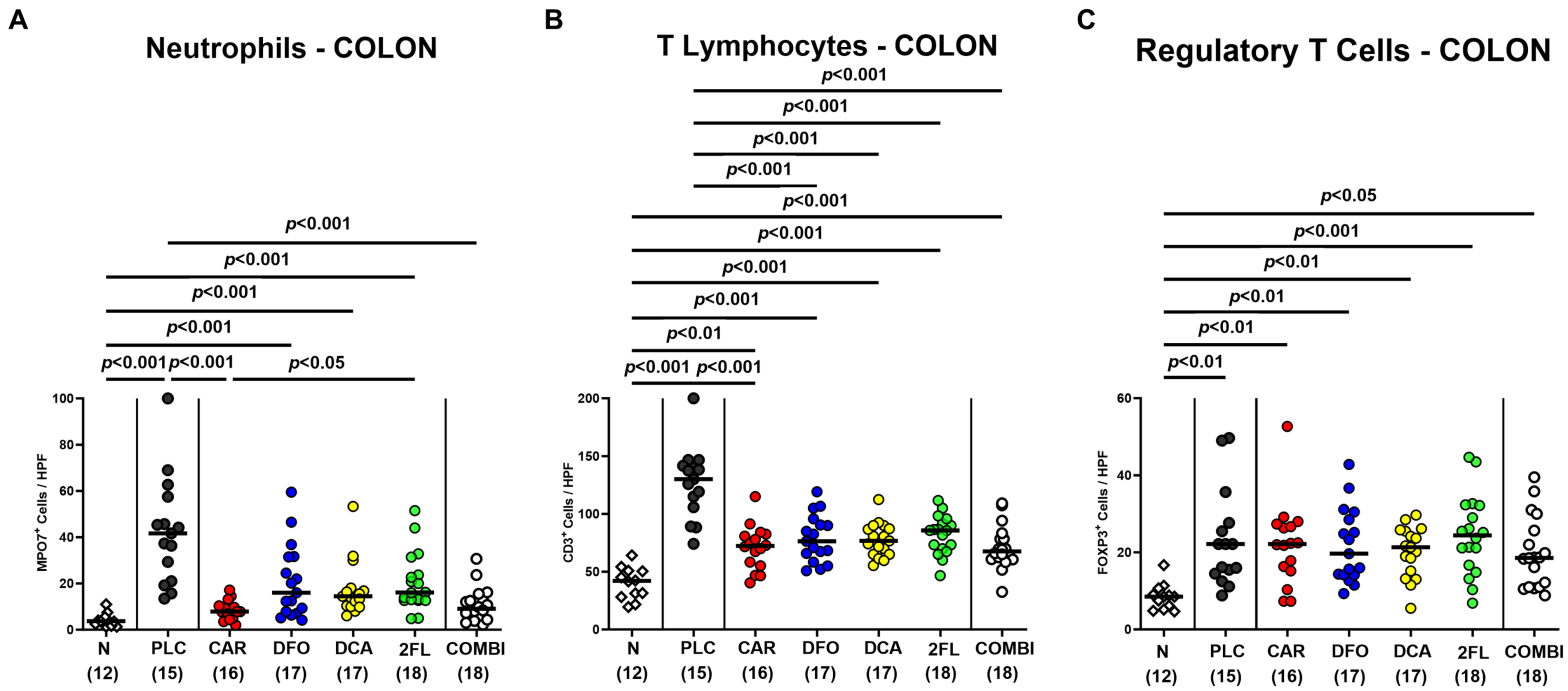
Immune cell subsets in the colon of hma IL-10^−/−^ mice following *C. jejuni* infection and treatment with carvacrol, deferoxamine, deoxycholic acid, and 2-fucosyl-lactose alone or all in combination. *C. jejuni* infected hma IL-10^−/−^ mice were perorally treated with synthetic carvacrol (CAR), deferoxamine (DFO), deoxycholic acid (DCA), 2-fucosyl-lactose (2FL), a combination of the four compounds (COMBI) or placebo (PLC) via the drinking water from day 2 until day 6 post-infection. **(A)** Neutrophils (MPO7^+^), **(B)** T lymphocytes (CD3^+^), and **(C)** regulatory T cells (FOXP3^+^) were enumerated in immunohistochemically stained colonic paraffin sections derived on day 6 post-infection (average numbers out of six representative high-power fields (HPF, 400 × magnification) per mouse). Naive (N) hma IL-10^−/−^ mice served as non-infected and untreated controls. Individual data pooled from three experiments, the medians (black bars), the numbers of included mice (in parentheses), and the significance levels (*p* values) determined by the Kruskal-Wallis test and Dunn’s post-correction **(A,C)** and the one-way ANOVA test with Tukey post-correction **(B)** are shown.

To test treatment impact on *C. jejuni* induced immune responses, we stained colonic paraffin sections with antibodies against surface markers of distinct innate and adaptive immune cells. On day 6 p.i., MPO7^+^ neutrophilic granulocytes were lower in the colonic mucosa and lamina propria of carvacrol and combination treated mice when compared to placebo counterparts (*p* < 0.001; Figure 6A). Remarkably, they did not even differ from uninfected controls (n.s. versus naive; Figure 6A). Irrespective of the treatment, *C. jejuni* infection resulted in colonic increases in adaptive CD3^+^ T lymphocytes and FOXP3^+^ regulatory T cells (*p* < 0.05–0.001 versus naive; Figures 6B,C). T cell numbers were, however, approximately 50% lower in the colon of all verum as compared to the control group on day 6 p.i. (*p* < 0.001; Figure 6B). Moreover, the combination of all four compounds did not further alleviate colonic T cell responses upon *C. jejuni* infection. Hence, oral carvacrol and combination treatment could diminish *C. jejuni* triggered immune responses.

### 3.6 Intestinal and extra-intestinal IFN-γ secretion in hma IL-10^−/−^ mice following *Campylobacter jejuni* infection and treatment with carvacrol, deferoxamine, deoxycholic acid, and 2-fucosyl-lactose alone or in combination

**Figure 7:**
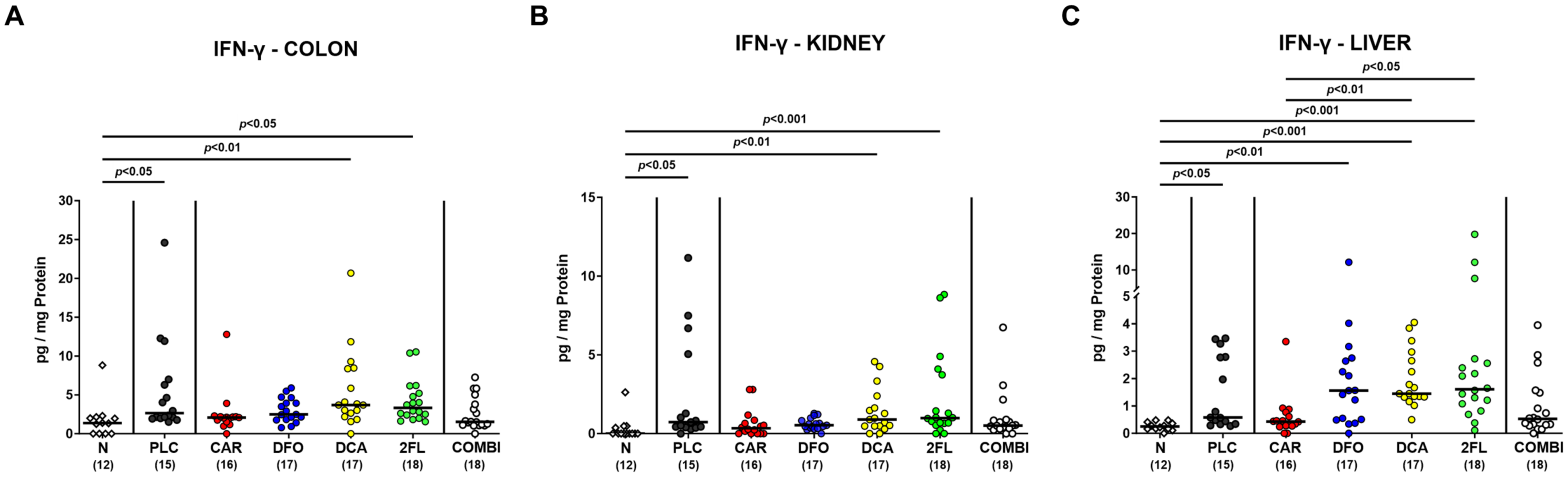
Intestinal and extra-intestinal IFN-γ concentrations in hma IL-10^−/−^ mice following *C. jejuni* infection and treatment with carvacrol, deferoxamine, deoxycholic acid, and 2-fucosyl-lactose alone or all in combination. *C. jejuni* infected hma IL-10^−/−^ mice were perorally treated with synthetic carvacrol (CAR), deferoxamine (DFO), deoxycholic acid (DCA), 2-fucosyl-lactose (2FL), a combination of the four compounds (COMBI) or placebo (PLC) via the drinking water from day 2 until day 6 post-infection. IFN-γ concentrations were determined in *ex vivo* biopsies taken from the **(A)** colon, **(B)** kidneys, and **(C)** liver on day 6 post-infection. Naive (N) hma IL-10^−/−^ mice served as non-infected and untreated controls. Individual data pooled from three experiments, the medians (black bars), the numbers of included mice (in parentheses), and the significance levels (*p* values) determined by the Kruskal-Wallis test and Dunn’s post-correction are shown.

*C. jejuni* infection resulted in enhanced IFN-γ secretion in colon and kidneys of placebo, deoxycholic acid and 2-fucosyl-lactose treated mice (*p* < 0.05–0.001; Figures 7A,B). In comparison, basal colonic and renal IFN-γ concentrations were measured in the carvacrol, deferoxamine, and combination treatment cohorts on day 6 p.i. (n.s. versus naive Figures 7A,B). This was also the case when determining IFN-γ levels in the liver of infected mice following carvacrol and combination treatment (n.s. versus naive Figure 7C). Hence, carvacrol and the combination treatment of hma IL-10^−/−^ mice dampens *C. jejuni* induced IFN-γ responses in colon and extra-intestinal compartments, as observed in kidneys and liver.

### 3.7 Pro-inflammatory cytokine secretion in the serum of hma IL-10^−/−^ mice following *Campylobacter jejuni* infection and treatment with carvacrol, deferoxamine, deoxycholic acid, and 2-fucosyl-lactose alone or in combination

**Figure 8:**
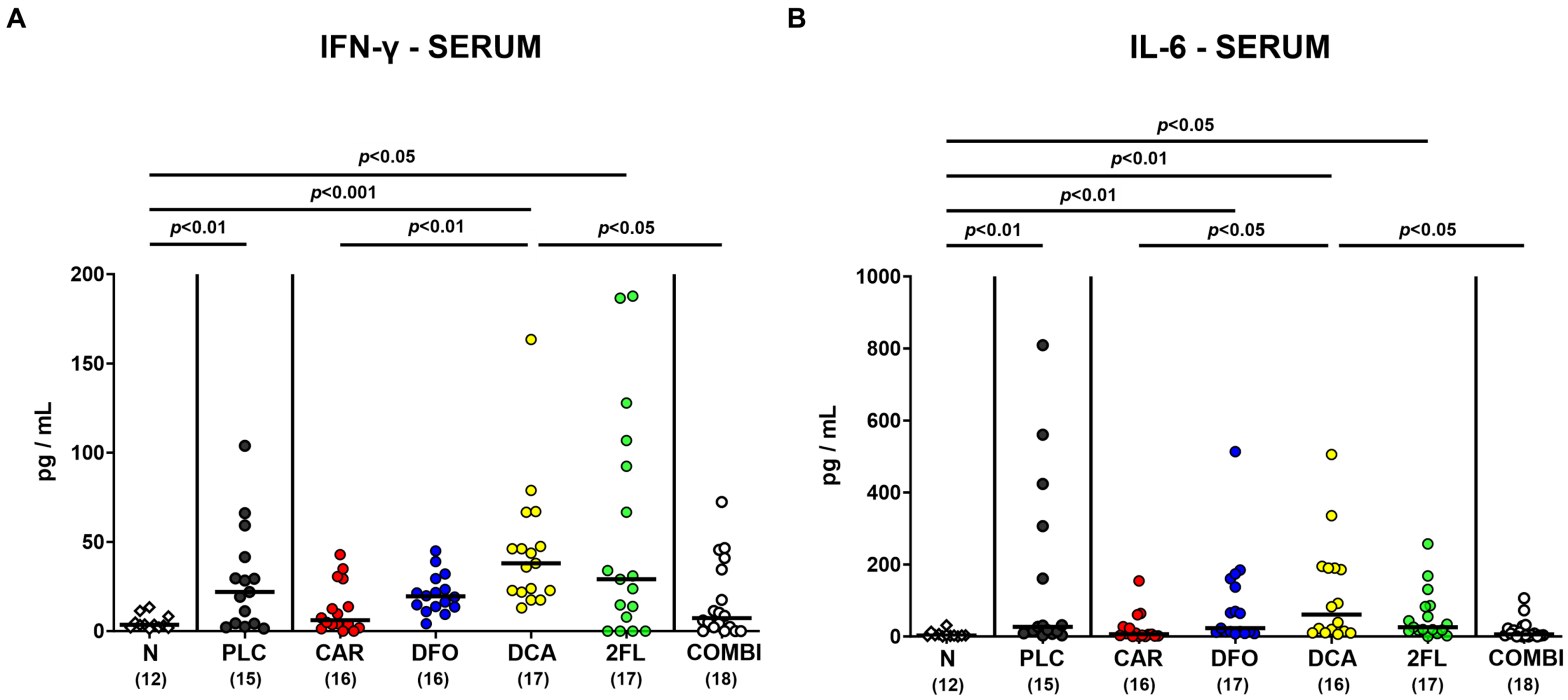
Systemic pro-inflammatory cytokine secretion in hma IL-10^−/−^ mice following *C. jejuni* infection and treatment with carvacrol, deferoxamine, deoxycholic acid, and 2-fucosyl-lactose alone or all in combination. *C. jejuni* infected hma IL-10^−/−^ mice were perorally treated with synthetic carvacrol (CAR), deferoxamine (DFO), deoxycholic acid (DCA), 2-fucosyl-lactose (2FL), a combination of the four compounds (COMBI) or placebo (PLC) via the drinking water from day 2 until day 6 post-infection. To assess systemic pro-inflammatory cytokine responses, **(A)** IFN-γ and **(B)** IL-6 concentrations were measured in serum samples taken on day 6 post-infection. Naive (N) hma IL-10^−/−^ mice served as non-infected and untreated controls. Individual data pooled from three experiments, the medians (black bars), the numbers of included mice (in parentheses), and the significance levels (*p* values) determined by the Kruskal-Wallis test and Dunn’s post-correction are shown. Outliers were excluded following identification by the Grubb’s test.

Further, we addressed whether the treatment regimens could even affect systemic *C. jejuni* induced cytokine responses. As for the kidneys, basal IFN-γ levels were measured in the serum of infected mice from the carvacrol, deferoxamine, and combination cohorts (n.s. versus naive: Figure 8A), which also held true for basal serum IL-6 concentrations determined in carvacrol and combination treated mice on day 6 p.i. (n.s. versus naive; Figure 8B). Hence, carvacrol and combination treatment of hma IL-10^−/−^ mice could alleviate systemic pro-inflammatory immune responses upon *C. jejuni* infection.

## 4 Discussion

For our current preclinical placebo-controlled intervention trial, we used (with respect to the gut microbiota) “humanized” IL-10^−/−^ mice that have been generated through triple oral human FMTs of secondary abiotic IL-10^−/−^ mice. Like all experimental systems, the hma IL-10^−/−^ mouse model has its own limitations, which need to be critically taken into consideration. For instance, distinct human bacterial phyla might have been affected by freezing and thawing of the human fecal donor suspensions. Furthermore, differences in engraftment of the human fecal transplants in the murine intestinal tract could affect the clinical and immunopathological course of infection (Shayya et al., 2023). Both, our cultural and molecular analyses of fecal samples taken 1-week following the human FMTs and hence, immediately before *C. jejuni* infection on day 0 revealed comparable fecal loads of the main intestinal commensal bacterial phyla in all six prospective treatment cohorts indicating similar intestinal bacterial conditions upon induction of campylobacteriosis. At the end of the observation period, no treatment regimens have affected *C. jejuni* loads in the colon, whereas on day 6 p.i. carvacrol treated hma IL-10^−/−^ mice displayed approximately two orders of magnitude lower pathogen loads in the ileum, but not the colon when compared to placebo counterparts. One can speculate that most of the administered carvacrol might have already been absorbed by enterocytes before reaching the colon and exerting a direct pathogen-lowering effect in the colonic lumen. Previous *in vitro* and *in vivo* studies have proven anti-*Campylobacter* directed effects of carvacrol, but with varying efficacies depending on the experimental model, the applied carvacrol doses, and the duration of treatment (Johny et al., 2010; van Alphen et al., 2012; Kelly et al., 2017; Szott et al., 2020). Nevertheless, one could argue that the 2-log reduction in the ileal pathogen loads alone was not sufficient to explain the observed clinical effects upon oral carvacrol application. An *in vitro* study by Upadhyay et al. revealed that carvacrol treatment resulted in reduced adhesion invasion, and translocation of *C. jejuni* (Upadhyay et al., 2017). These results might explain the disease alleviating effect of oral carvacrol without pronounced antibacterial effects directed against *C. jejuni* infection *in vivo.* Nevertheless, other aspects such as the metabolization of carvacrol by members of the intestinal microbiota or direct immune-modulatory effects of the compound may have contributed to the better outcome of *C. jejuni* induced disease in treated mice.

In line with our results, even prophylactic application of deferoxamine in infected IL10^−/−^ mice did not lower intestinal *C. jejuni* loads (Bereswill et al., 2022). Unexpectedly, deoxycholic acid and 2′-fucosyl-lactose did not exert antibacterial effects against *C. jejuni*, although numerous *in vitro* and *in vivo* studies have reported antimicrobial effects against various Gram-negative bacteria such as *Pseudomonas aeruginosa*, *E. coli*, *Salmonella* spp., and especially *Campylobacter*, for both substances (Yu et al., 2016; Gourley et al., 2017; Negretti et al., 2017; Alrubaye et al., 2019; Facinelli et al., 2019).

The lack of anti-*Campylobacter* effects of exogenous deoxycholic acid and 2′-fucosyl-lactose in our preclinical trial might be explained by concentrations in the drinking solutions that were below the measured MICs. Even though the concentrations of carvacrol, deferoxamine and all four combined compounds in the drinking solutions were exceeding the MIC of respective compounds measured *in vitro*, the treated mice harbored the enteropathogen at numbers that did not differ from those determined in placebo counterparts. This unexpected result might be explained due to mixing and dilution effects of the applied substances with secretory intestinal fluids, which have potentially resulted in a rather subtle or even absent antibacterial effect. This is further supported by the fact that mice do not exhibit continuous drinking behaviors during day and night.

Despite the high gastrointestinal pathogen burdens, carvacrol and the quadruple combination treatment did not only result in less severe diarrheal symptoms in *C. jejuni* infected hma mice, but also prevented diarrhea in over 80% of infected mice that did not present any changes in stool consistency on day 6 p.i. This could be confirmed by our previous study showing potent anti-diarrheal effects of prophylactic oral carvacrol in *C. jejuni* infected secondary abiotic IL10^−/−^ mice (Mousavi et al., 2020). Furthermore, carvacrol was shown to inhibit the diarrheal toxin production by *Bacillus cereus in vitro* (Ultee et al., 2002) and to alleviate *Clostridioides difficile* associated diarrheal symptoms in mice (Mooyottu et al., 2017).

The marked improvement in diarrheal symptoms following carvacrol and combination treatment of hma IL-10^−/−^ mice was accompanied by less pronounced microscopic inflammatory sequelae of *C. jejuni* infection like histopathological changes and apoptotic epithelial cell responses in the large intestine. In line with these findings, carvacrol strongly inhibited caspase-dependent apoptosis and down-regulated the mTOR-Signaling *in vitro* (Banik et al., 2019), whereas prophylactic carvacrol application attenuated *C. jejuni*-induced apoptosis in colonic epithelia of infected secondary abiotic IL-10^−/−^ mice (Mousavi et al., 2020). Besides carvacrol, also oral deferoxamine and 2′-fucosyl-lactose alone diminished apoptotic colonic cell responses upon *C. jejuni* infection. In line with our findings, a study in adult rats showed that iron overload increased caspase-3 reactivity whereas, conversely, deferoxamine treatment ameliorated iron-induced intestinal apoptosis and inflammation (El-Sheikh et al., 2018). Furthermore, 2-fucosyl-lactose was shown to protect small intestinal epithelial cells against 5-fluorouracil-induced apoptosis (Zhao et al., 2022).

In addition to alleviated diarrheal symptoms and colonic epithelial cell apoptosis, oral carvacrol and quadruple combination treatment dampened pro-inflammatory immune cell responses in *C. jejuni* infected hma mice as indicated by attenuated accumulation of neutrophils and T lymphocytes in the colonic mucosa and lamina propria. Moreover, *C. jejuni* induced colonic T cell responses were decreased upon deferoxamine, deoxycholic acid, and 2-fucosyl-lactose application alone. In support, our previous studies revealed that *C. jejuni* infected IL-10^−/−^ mice pre-treated with carvacrol or deferoxamine exhibited lower numbers of T lymphocytes in the large intestines, as compared to placebo controls (Mousavi et al., 2020; Bereswill et al., 2022). Additionally, 2′-fucosyl-lactose pretreatment was shown to reduce CD3^+^ T cell infiltration of the colon in *C. jejuni* infected wildtype mice (Yu et al., 2016). When assessing pro-inflammatory cytokine secretion in the infected large intestines, mice from the carvacrol, the deferoxamine, and the combination cohorts exhibited colonic IFN-γ concentrations that did not differ from those determined in naive mice. The anti-inflammatory effects of the applied compounds could also be observed beyond the intestinal tract given only basal IFN-γ concentrations measured in the kidneys, the liver, and even in the serum of infected mice from the carvacrol, the deferoxamine, and the combination treatment cohorts. In support, carvacrol treatment was shown to down-regulate IFN-γ expression in splenocytes of asthmatic mice (Kianmehr et al., 2016) and to decrease IFN-γ and IL-6 secretion in mice suffering from multiple sclerosis (Mahmoodi et al., 2019). A very recent study revealed that oral treatment with the iron chelating compound deferasirox alleviated acute dextran sulfate sodium (DSS) induced colitis that was accompanied by decreased IFN-γ serum concentrations (Wu et al., 2023), further underscoring the immune-modulatory effects of iron deprivation in acute intestinal inflammation including campylobacteriosis as shown for deferoxamine in our present and earlier studies (Bereswill et al., 2022).

Although previous reports have highlighted that both deoxycholic acid and 2-fucosyl-lactose attenuated alleviated intestinal inflammation including *C. jejuni* induced colitis in mice (Yu et al., 2016; Sun et al., 2018), no overt immune-modulatory effects of both substances were observed in our present trial. These discrepancies might be due to differences in experimental settings including dosage and duration of treatment, gut microbiota composition, genetic background, and animal husbandry, for instance.

Furthermore, given the here applied 4-day therapeutic treatment regimens, the disease-alleviating effects of respective compounds alone or in combination might have been more prominent after longer application periods starting before infection (i.e., prophylactic regimen). This might be addressed in future investigations.

Finally, the here applied “humanized” IL-10^−/−^ mouse model provides a useful experimental tool (i) to dissect the interactions between *C. jejuni* on the pathogen side and the immune system as well as the commensal (murine or human) gut microbiota on the host side and (ii) to test defined molecules and compounds for their potential disease-alleviating including anti-pathogenic and immune-modulatory properties (Shayya et al., 2023).

## 5 Conclusion

Our placebo-controlled preclinical intervention study provides evidence that oral treatment with carvacrol, deferoxamine, deoxycholate, and 2-fucosyl-lactose exerts disease-alleviating effects in acute campylobacteriosis. All substances are approved by the U.S. Food and Drug Administration (Food and Drug Administration, 2023) and have proven to exert disease-alleviating effects in acute campylobacteriosis. In particular, the highly potent intestinal, extra-intestinal, and systemic anti-inflammatory properties of the non-toxic natural compounds might point towards a promising alternative in the fight of acute campylobacteriosis with antibiotics-independent treatment options.

## Data availability statement

The original contributions presented in the study are included in the article/Supplementary material, further inquiries can be directed to the corresponding author.

## Ethics statement

The animal study was approved by “Landesamt für Gesundheit und Soziales”, LaGeSo, Berlin. The study was conducted in accordance with the local legislation and institutional requirements.

## Author contributions

SM: Formal analysis, Investigation, Visualization, Writing – original draft. MF: Formal analysis, Investigation. KD: Investigation, Writing – review & editing. RB: Investigation. SB: Conceptualization, Funding acquisition, Supervision, Writing – review & editing. MMH: Conceptualization, Funding acquisition, Investigation, Supervision, Validation, Writing – original draft.
