# Supplementary material for: Oral treatment of human gut microbiota associated IL-10−/− mice suffering from acute campylobacteriosis with carvacrol, deferoxamine, deoxycholic acid, and 2-fucosyl-lactose
Source: Front Microbiol. 2024 Jan 25;15:1290490. doi: 10.3389/fmicb.2024.1290490 (PMC10854223; doi:10.3389/fmicb.2024.1290490)
Supplement: Supplementary file 1 [file Data_Sheet_1.docx]

Supplementary Figures


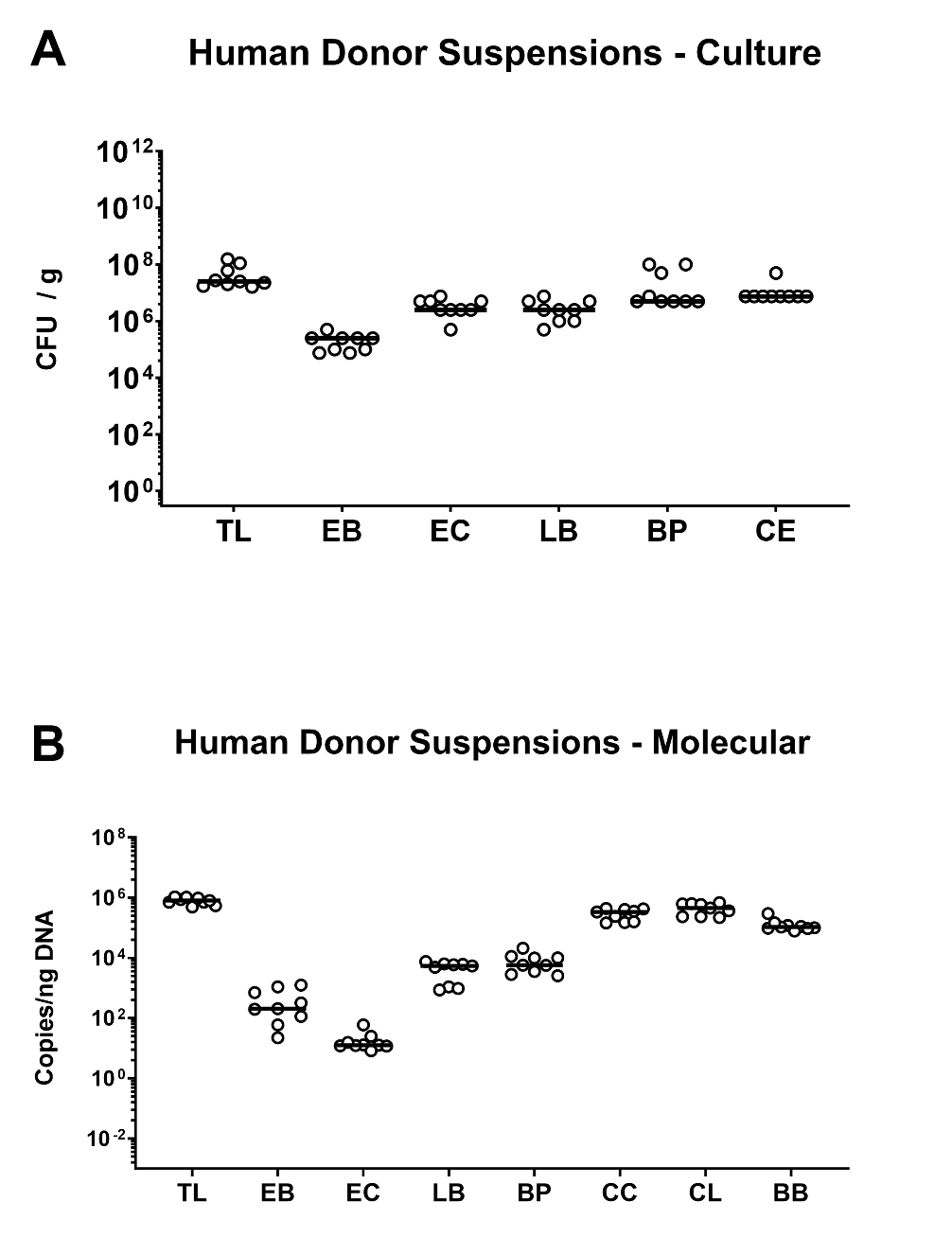


Supplementary Figure 1. Gut microbial communities in human fecal donor suspensions. A week before *C. jejuni* infection, secondary abiotic IL-10^-/-^ mice (that had been generated by antibiotic pretreatment) were subjected to human fecal microbiota transplantation on three consecutive days by oral gavage (i.e., on day (d)-7, d-6, and d-5). The gut microbial communities in the human fecal donor suspensions were quantitatively assessed by (A) culture (expressed as colony-forming units per gram (CFU / g) and (B) culture-independent, molecular analyses (expressed as gene copies per ng DNA). Data pooled from three experiments and medians are shown. TL, total load; EB, *enterobacteria*; EC, enterococci; LB, lactobacilli; BP, *Bacteroides/Prevotella* species; CE, *Clostridium/Eubacterium* species; CC, *Clostridium coccoides* group; CL, *Clostridium leptum* group; BB, bifidobacteria. Medians are indicated by black bars.


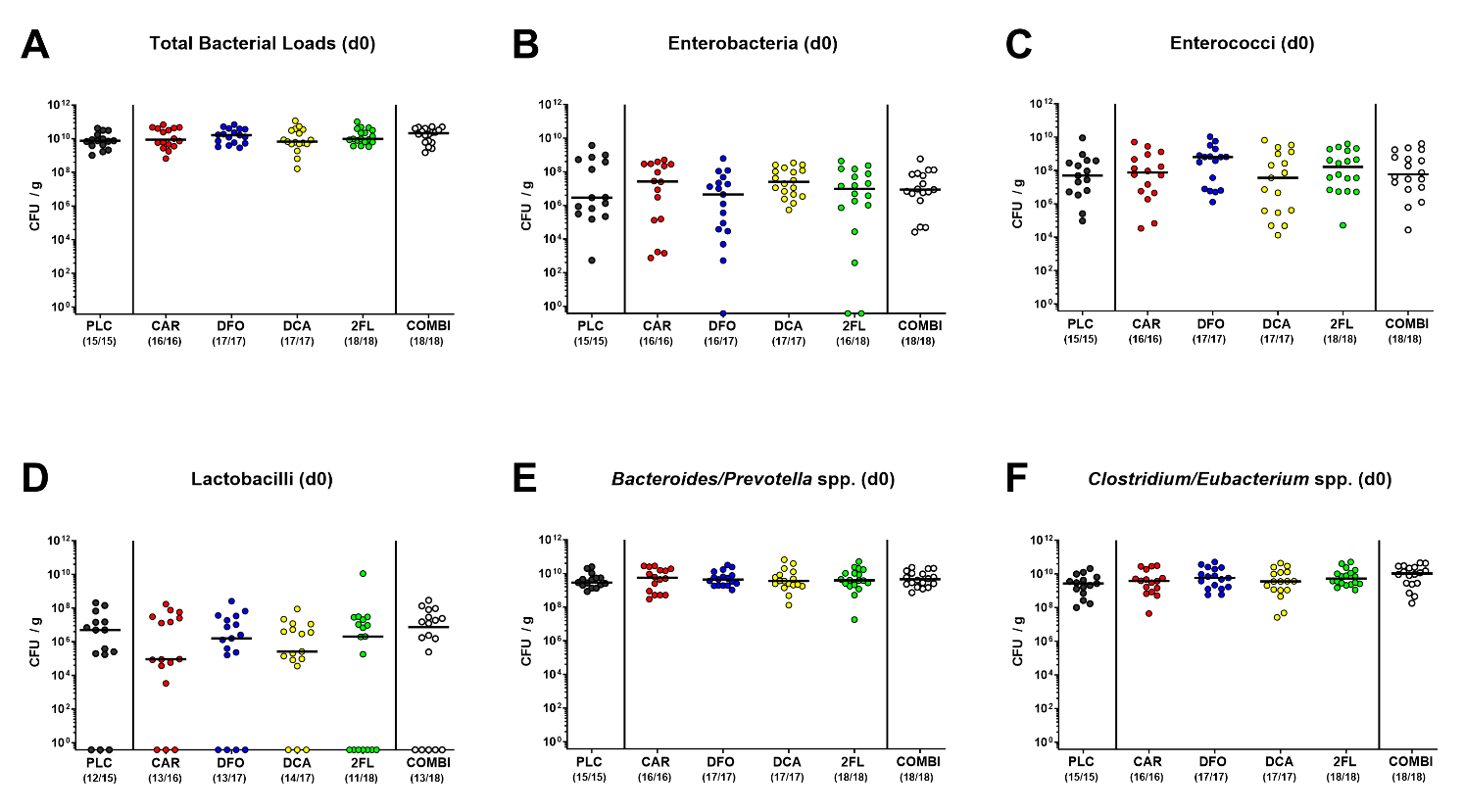


**Supplementary Figure 2.** **Cultural analysis of the fecal microbiota composition in hma IL-10^-/-^ mice immediately before *C. jejuni* infection**. Hma IL-10^-/-^ mice were generated by oral human fecal microbiota transplantation of secondary abiotic mice on three consecutive days (i.e., on day (d)-7, d-6, and d-5). Immediately before *C. jejuni* infection on d0, the fecal microbiota composition was surveyed in the hma mice from the prospective treatment cohorts (PLC, placebo; CAR, carvacrol; DFO, deferoxamine; DCA, deoxycholic acid; 2FL, 2-fucosyl-lactose; COMBI, combination) by culture (see methods) and the **(A)** total bacterial loads, **(B)** enterobacteria, **(C)** enterococci, **(D)** lactobacilli, **(E)** *Bacteroides/Prevotella* species (spp.), and **(F)** *Clostridium/Eubacterium* spp. expressed an colony-forming units per gram (CFU / g). Medians (black bars) and numbers of culture-positive mice out of the total number of analyzed animals (in parentheses) are indicated.


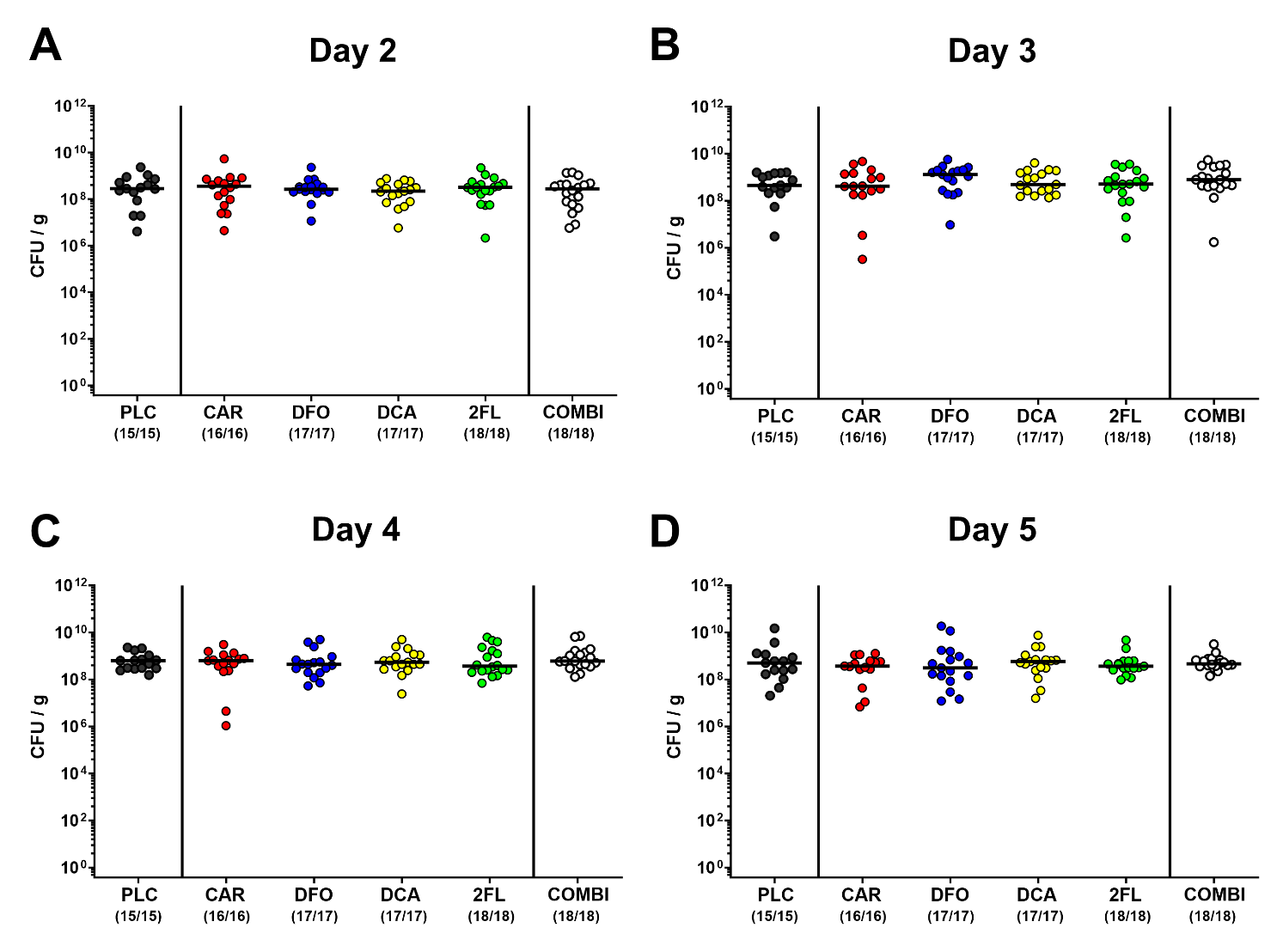


**Supplementary Figure 3. Establishment of *C. jejuni* in the intestinal tract of infected hma IL-10^­‑/-^ mice over time following treatment with carvacrol deferoxamine, deoxycholic acid and 2-fucosyl-lactose alone or all in combination**. Hma IL-10^-/-^ mice were orally infected with *C. jejuni* strain 81-176 on days 0 and 1. From day 2 until day 6 post-infection, mice were treated with synthetic carvacrol (CAR), deferoxamine (DFO), deoxycholic acid (DCA), 2-fucosyl-lactose (2FL), a combination of all four compounds (COMBI) or placebo (PLC) via the drinking water. The establishment of *C. jejuni* in the intestinal tract over time were quantitatively assessed in fecal samples taken on **(A)** day 2, **(B)** day 3, **(C)** day 4, and **(D)** day 5 post-infection by culture and expressed as colony-forming units per gram (CFU / g). Individual data pooled from three experiments, medians (black bars), and the numbers of culture-positive mice out of the total number of analyzed animals (in parentheses) are shown.


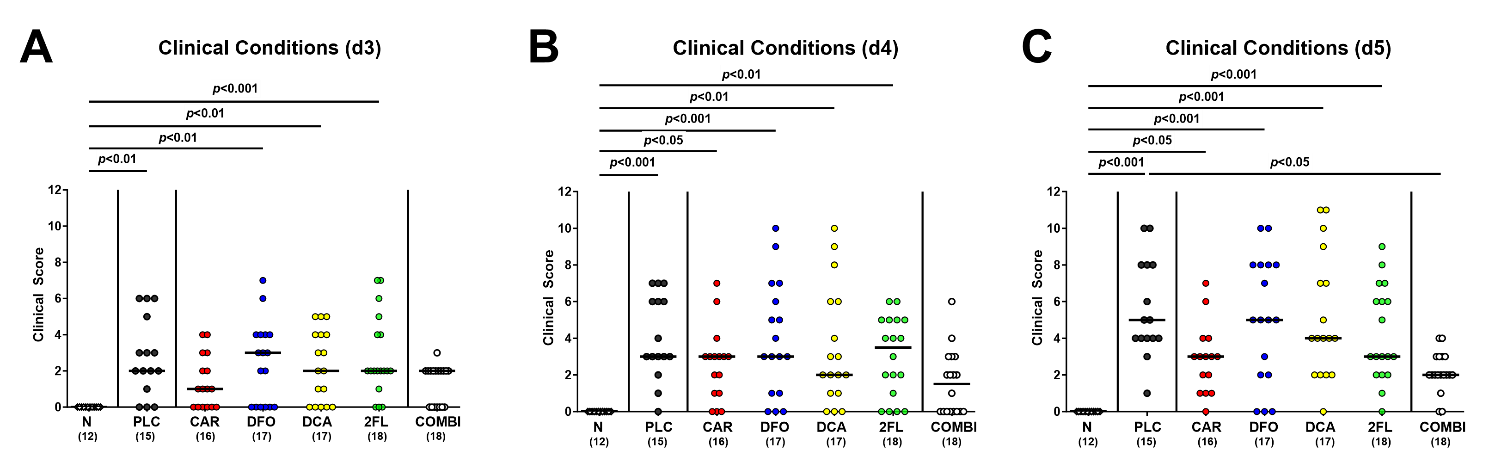


**Supplementary Figure 4. Clinical conditions of infected hma IL-10^-/-^ mice over time following treatment with carvacrol, deferoxamine, deoxycholic acid and 2-fucosyl-lactose alone or all in combination**. Hma IL-10^-/-^ mice were orally infected with *C. jejuni* strain 81-176 on day (d)0 and d1. From d2 until d6 post-infection, mice were treated with synthetic carvacrol (CAR), deferoxamine (DFO), deoxycholic acid (DCA), 2-fucosyl-lactose (2FL), a combination of all four compounds (COMBI) or placebo (PLC) via the drinking water. The clinical conditions of mice were quantitatively assessed with a clinical score on **(A)** d3, **(B)** d4, and **(C)** d5 post-infection. Naive (N) hma IL-10^-/-^ mice served as non-infected and untreated controls. Individual data pooled from three experiments, the medians (black bars), the numbers included mice (in parentheses), and the significance levels (*p* values) determined by the Kruskal-Wallis test and Dunn’s post-correction are shown.
